# Effect of silver nanoparticles foliar application on the nutritional properties of potato tubers

**DOI:** 10.1038/s41598-024-73096-2

**Published:** 2024-09-18

**Authors:** Krzysztof M. Tokarz, Tomasz Mazur, Monika Hanula, Wojciech Makowski, Piotr Zawal, Roman J. Jędrzejczyk, Konrad Szacilowski, Stanisław Mazur, Wojciech Wesołowski, Barbara Tokarz

**Affiliations:** 1 Department of Botany, Physiology and Plant Protection, Faculty of Biotechnology and Horticulture, University of Agriculture in Krakow, al. 29 Listopada 54, Kraków, 31-425 Poland; 2 Academic Centre for Materials and Nanotechnology, AGH University of Krakow al. A, Mickiewicza 30, Kraków, 30-059 Poland; 3 Institute of Human Nutrition Sciences, Warsaw University of Life Sciences, Nowoursynowska 159c Street 32, Warsaw, 02-776 Poland; 4 Plant-Microorganisms Interaction Group, Malopolska Centre of Biotechnology, Jagiellonian University, ul. Gronostajowa 7A, Kraków, 30-387 Poland; 5 Krakow, Poland

**Keywords:** Ag nanoparticles, Elements, Phenolic compounds, Radical scavenging activity, Sugars, Vitamin C, Biotechnology, Plant sciences

## Abstract

The aim of presented study was to test nutritional properties of potato tubers and silver ions accumulation pattern after foliar application of silver nanoparticles (AgNPs) during potato vegetation. Potato plants were sprayed with different concentration of Ag nanoparticles (0.1, 1.0 and 10 mg·dm^−3^) synthesized with incorporation with sodium dodecyl sulphate (SDS) and sodium citrate as stabilizing agent. The lowest amounts of silver ions were transported to the tubers after spraying with AgNPs synthesized with SDS, rather than with citrate. Nevertheless silver ions accumulation in tubers was negligible. SDS method of synthesis was more favourable in terms of nutritional properties of potato tubers. The highest tested concentration of AgNPs_SDS had a favourable effect on a variety of macro- and micronutrients, ascorbic acid and soluble sugars. In turn, lower concentrations of AgNPs_SDS increased the content of phenolic compounds and free radical scavenging efficiency of tubers. These correlations were also confirmed by Principal Component Analysis.

**Supplementary Information:**

The online version contains supplementary material available at 10.1038/s41598-024-73096-2.

## Introduction

Potato (*Solanum tuberosum* L.) is the third most important food crop in the world after rice and wheat in terms of human consumption, and the size of the global crop was 17 mln ha in 2020, while yields were 217, 688 hg/ha^1^. Potato is not only a rich source of energy but has many valuable nutritional compounds, including proteins, mainly patatin reach in exogenous amino acids (lysine, phenylalanine, leucine, isoleucine threonine, valine, )^2^, dietary fiber, vitamins C, B1, B6, and elements (potassium, magnesium, iron, zinc, phosphorus), which are important for human health. Moreover, the potato tubers accumulate phytosterols and secondary metabolites, such as anthocyanins or phenols. Compounds of phytochemicals help to reduce the risk of diseases such as cardiovascular diseases (via reduced serum LDL-cholesterol levels), cancers, and inflammatory (via compounds, which are responsible for antioxidant activity)^3–5^.

Nanotechnology solutions using metal nanoparticles are gaining popularity, mostly due to their unique properties: high surface/volume ratio, the potential for surface modification, thermal activities, antibacterial and antioxidant activity^6^. Silver nanoparticles (AgNPs) in particular are known for their antimicrobial properties, which is why they have become an alternative to traditional plant protection products^7,8^. This is particularly important nowadays, when efforts are being made to reduce the use of chemical protection products^9^. Despite the antimicrobial properties of protectants, they not only affect the physiology of pathogens eliminating the pathogenic threat but also affect the physiological processes of the plant. Previous reports indicate that, depending on the plant species and its morphological and physiological conditions, the effect of AgNPs can be either positive, resulting in plant growth and development, or negative – inducing DNA damages, causing decrease in the activity of the antioxidant system or breakdown of the cytoplasmic membrane^10^. Moreover, nanoparticles also affect the nutritional properties of edible plant parts^11–16^. For example, silver nanoparticles significantly altered the nutritional values of: peppers fruit (mineral nutrients, crude proteins, lipids and carbohydrates)^12^, pea seeds (proteins and carbohydrates)^13^, wheat grain (amino acids, proteins, Fe, Cu and Zn)^14^ cabbage leaves (amino acids and Fe)^15^ and radish sprouts (Ca, Mg, B, Cu, Mn, Zn, carbohydrates, lignin and lipids)^16^. Noting that the changes were both positive - an increase in the content of various components (peppers, cabbage, peas) and negative - a decrease (wheat, radish)^12–16^. However, no literature describes changes in the nutritional value of potato tubers due to foliar application of AgNPs until now.

The effect of AgNPs depends on their dose and physicochemical properties that are different depending on the method of synthesis^17^. Different synthetic routes allow obtaining nanoparticles of various sizes^17^. The dimensions of particles influence their antimicrobial activity: the smaller particles, the greater antimicrobial effect^18^. Furthermore, the AgNPs effect depends also on the method of nanoparticles application. The possibility of penetration, speed of transport, nature of interaction with the plant and the place of Ag accumulation are of key importance in the context of the potential of direct use of AgNPs in agricultural plant protection treatments. Therefore, it seems that a properly selected dose and structure of the nanoparticles can be of great importance in the case of crops production. Considering food safety, it is extremely important to know where metals accumulate and how a particular protectant affects the nutrient compounds in the edible parts of the plant.

In current work, we have hypothesized that application of AgNPs in appropriate sizes and concentrations in potato cultivation allows for Ag accumulation only in above-ground parts of the plant and prevents further Ag relocation to tubers, while improving the nutritional properties of the tubers. The research aimed to examine: (1) nutritional properties of potato tubers and (2) accumulation pattern of Ag ions in potato shoots and tubers after foliar application of AgNPs during potato vegetation.

This paper, describes the relationship between methodology of obtaining AgNPs and Ag accumulation in plants and tubers and the nutritional properties of tubers in field potato cultivation.

## Materials and methods

### Nanoparticles synthesis

For the experiments, two synthetic procedures were incorporated, based on literature findings (see below). All of the reagents were purchased from commercial vendors and used without prior purification: AgNO_3_ (POCh, analytical grade), hydrazine monohydrate (Alfa Aesar), sodium citrate (Sigma Aldrich) and sodium dodecyl sulfate (SDS) (Sigma Aldrich, technical grade). In each synthesis hydrazine was used as reducing agent.

The first synthetic route^19^ used sodium dodecyl sulfate as capping agent, which stabilizes the nanoparticles and prevents them from agglomeration – thus latter samples will be denoted AgNP_SDS. The synthetic procedure was as follows: colloidal silver particles were synthesized by the reduction of AgNO_3_ by hydrazine in the presence of SDS. Firstly, 95 cm^3^ of 1∙10^−2^ mol·dm^−3^ AgNO_3_ solution was mixed with 150 cm^3^ of 1∙10^−1^ mol·dm^−3^ SDS – using magnetic stirrer for 30 min set for 400 rpm. Next, hydrazine solution (0.02 cm^3^ of monohydrate in 20 cm^3^ of distilled water) was added dropwise, until the solutions turned light green and some debris precipitated. The whole reaction was conducted in ambient conditions (room temperature = 23 °C, constant mixing) for 30 min. The supernatant and precipitate were sonicated for 5 min and centrifuged at 8000 rpm using MPW-260R centrifuge (MPW MED. INSTRUMENTS, Warsaw, Poland). This procedure was repeated twice, leaving all of the permanently agglomerated nanoparticles in precipitate, which was later discarded and the supernatant was collected. The remaining supernatant with dispersed and separated nanoparticles was used to prepare three sets of working solutions. The latter were prepared from 37, 3.7 and 0.37 cm^3^ of the original solutions with the addition of water up to total volume of 500 cm^3^. For the most concentrated solutions Ag concentration was estimated (assuming 50% efficiency of centrifugation separation for the original solutions) to be 10 mg·dm^−3^. The samples were denoted, from most to least concentrated one: AgNP_SDS_10, AgNP_SDS_1 and AgNP_SDS_0.1 respectively, and subsequently used in field experiments. The measured pH values of the solutions were 9 (AgNP_SDS_10) and 8 (AgNP_SDS_1 and AgNP_SDS_0.1.)

The second synthesis was also an altered literature protocol^17^ which 275 cm^3^ of 1∙10^−3^ mol·dm^−3^ AgNO_3_ was mixed with 25 cm^3^ of 1% sodium citrate (C_6_H_5_O_7_Na_3_) and subsequently mixed on a magnetic stirrer (400 rpm, 10 min). While still mixing, 3 cm^3^ of hydrazine solution (0.02 cm^3^in 20 cm^3^ of distilled water) was added dropwise and mixed for another 30 min in room temperature. The as-obtained solution was cloudy and yellow. To remove the precipitate, which was constituted by agglomerated Ag NPs, Ag microparticles and other particulate matter, the solution was sonicated for 5 min and centrifuged at 8000 rpm – process was repeated twice. The decanted precipitate was further discarded, and the remaining solution was used for the preparation of working solutions. Similar to previously described protocol solution was used to prepare three sets of working solutions. The latter were prepared from 103, 10.3 and 1.03 cm^3^ of AgNP_citr solution were mixed with water up to total volumes of 500 cm^3^. Sample names were AgNP_citr_10 (Ag concentration equal to 10 mg·dm^−3^), AgNP_citr_1 and AgNP_citr_0.1, respectively. The measured pH values of the solutions were 6 (AgNP_citr_10) and 5 (AgNP_citr_1: and AgNP_citr_0.1).

Shortly after synthetic stages, physicochemical characterization was initiated by UV-Vis spectra, which were registered on Agilent 8453 spectrophotometer (Agilent Technologies, Inc., Santa Clara, CA, USA) in quartz cell of 10 mm optical path length. Average size distribution was measured by dynamic light scattering technique (DLS) on Litesizer 500 and Zetasizer Nano (Anton Paar GmbH., Graz, Austria). Alternatively size distribution and morphology of AgNPs used in field experiments were also investigated by FEI Versa 3D FEG scanning electron microscope (Thermo Fisher Scientific inc., Waltham, MA, USA).

### Pot experiment

The pot experiment was conducted in 2019 (March -June) in controlled conditions of greenhouse of University of Agriculture in Krakow located in Prądnik Bialy district of Krakow (50°08’N, 19°95’E).Plant material comprised of potato (*Solanum tuberosum* L.) variety ‘Tajfun’ (R 1412, COBORU Research Centre for Cultivar Testing). Tubers for planting were obtained from Pomeranian-Mazurian Potato Breeding Company Ltd. (Strzekęcino, Poland), which was also responsible for formal identification of the plant material. ‘Tajfun’ variety is edible, medium-early and belongs to the B-BC consumer type (slightly concise and floury)^20^. Seed potato tubers were planted in 7.5 dm^3^ pots, filled with loam soil. Polifoska 10-5-15-5-14 (N, P, K, Mg, S) fertilizer (Grupa Azoty, Police, Poland) was applied into the soil at a dose of 0.35 g∙kg^−1^ soil prior planting. Throughout the entire cultivation period, this was the sole mineral fertilization of the plants. The plants were irrigated using a drip irrigation system with tap water at a rate of approximately 200 to 500 cm^3^ per pot.

The first AgNP spraying of the potato plants was carried out 4 weeks after the plants emergence in potato vegetation stage: BBCH 51–55^21^, subsequent spraying was carried out every 2 weeks (BBCH 65–69 and BBCH 81–85). In total, three sprays were performed. Each combination (control-without spraying, SDS_10, SDS_1, SDS_0.1, citr_10, citr_1, citr_0.1) were established in three replicates, each replicate consisted of ten plants (ten pots). We tested 2 factors: (1) nanoparticle synthesis methods (with SDS or citrate) and nanoparticle concentration (0.1, 1 and 10 mg-dm^−3^) and (2) parts of the tuber (pith, core and periderm). The experiment was carried out in two replicates.

**Fig. 1:**
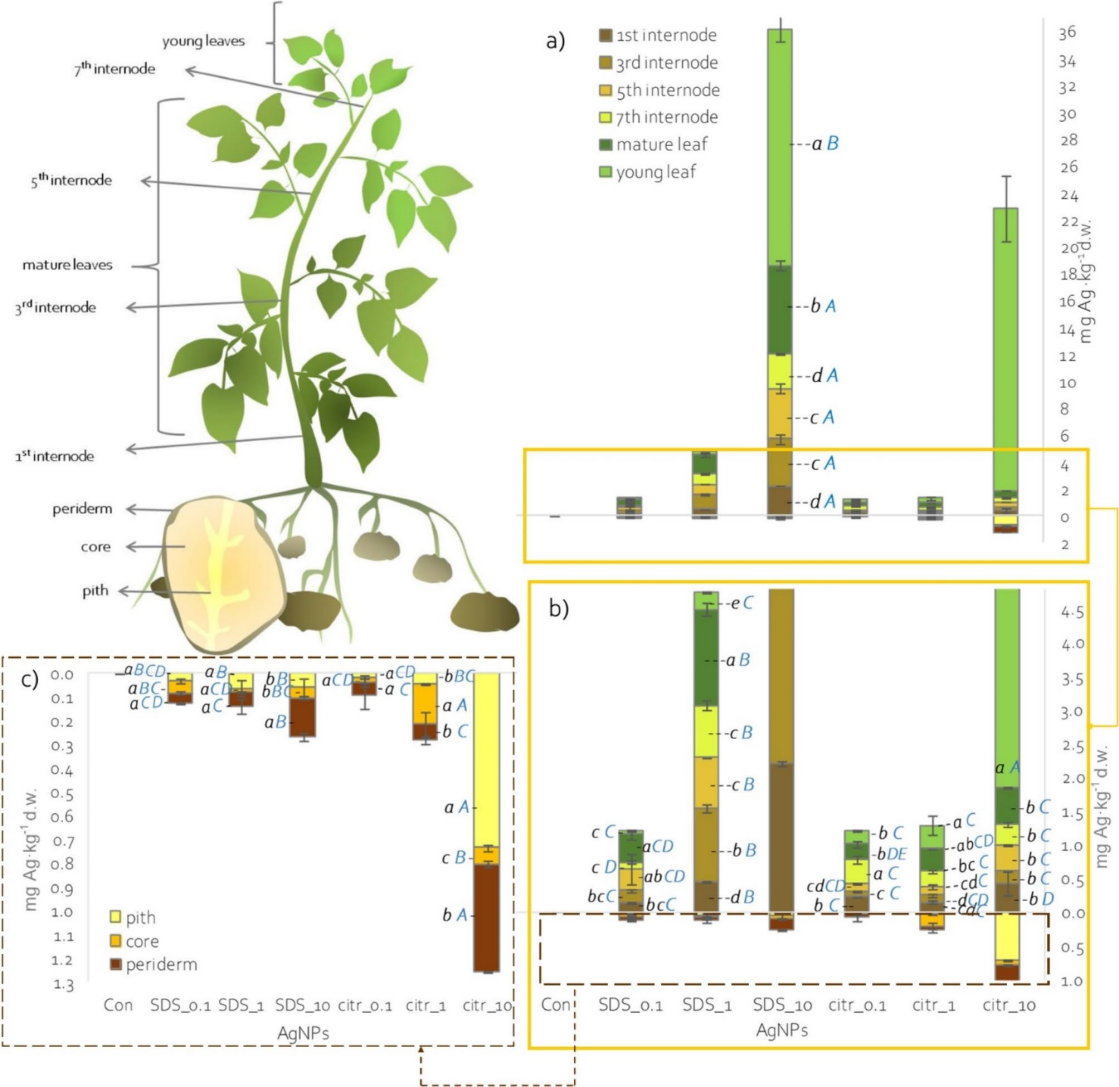
Content Ag^+^ ions in shoots (**a**-**b**) and tubers (**c**) of potato plants depending on applied spraying solutions; different lowercase letters – statistically significant differences between shoot parts and tuber parts within single spraying solution at p ≤ 0.05; different uppercase letters - statistically significant differences between spraying solutions within single plant part at p ≤ 0.05; n = 3.

After 2 weeks from the last spraying (BBCH 91–93), plant material samples were collected. From each combination nine plants were collected (three plants from each replicate). After a thorough cleaning of plants with tap and deionized water, the plants were divided. From the aboveground plant part, young and mature leaves and first, third, fifth and seventh internode (counting from the ground) were collected. Tubers were divided into periderm, core and pith fragments (Fig. 1).

### Plant material analyses

#### Analyses of elements’ concentration in shoots and tubers

About 200 mg of plant material (young leaf, mature leaf, 1st, 3rd, 5th, 7th internode, periderm, core and pith of tuber) were subjected to microwave (speedwave ENTRY, Berghof, Eningen unter Achalm, Germany) assisted acid digestion in 5 cm^3^ of HNO_3_ and 1.65 cm^3^ of 30% H_2_O_2_^22^. After digestion the solutions were made up to 25 cm^3^ with Milli-Q^®^ water. The samples of shoot and tuber parts were analyzed for total Ag concentration. Samples of tuber’ periderm, core and pith were analyzed also for total K, Na, Fe and Zn concentration. Flame atomic absorption spectrometry equipped with a CSX 260 auto-sampler (Thermo Scientific iC 3000, Waltham, MA, USA), was used to determine metal concentration. External standard calibration method was used.

#### Analyses of tuber composition and antioxidant properties

##### Estimation of L-ascorbic acid content

The Tillmans method of oxidometric titration was used to determine L-ascorbic acid (LAA) content^23^ in tuber tissues (periderm, core and pith fragments). The plant material (12.5 g f.w.) was homogenized in 50 cm^3^ of 1% acetic acid as an acidity regulator. After 30 min, the extract was titrated with Tillman’s reagent (2,6-dichlorophenolindophenol) until a pink color persisted for 30 s. The L-ascorbic acid content of the sample was calculated from the amount of Tillman’s reagent used for titration and expressed as g of L-ascorbic acid per kg of f.w.

##### Estimation of soluble sugar content

Soluble sugar content was determined using the anthrone reagent method^24^. Fresh plant material (1 g) was extracted with 70% ethanol in conical flask heated under a reflux condenser for half an hour from the start of alcohol boiling with the sample. Alcoholic extracts were diluted with deionized water. 2 cm^3^ of the aqueous solution were mixed with anthrone reagent solution (1 g anthrone in 500 cm^3^ 72% H_2_SO_4_) and heated for 10 min at 95 °C. Reaction was terminated in cold water. The absorbance of the solutions was measured at 625 nm using Unico 2804 UV spectrophotometer (Suite E Dayton, Newark, NJ, USA). A glucose calibration curve was used to calculate the content of sugars (g·kg^−1^ f.w.).

Estimation of phenols content and radical scavenging activity.

Phenolics compounds were determined in tuber tissues (periderm, core and pith fragments). The total content of phenolics compounds was measured spectrophotometrically according to Swain and Hillis^25^ with some modifications. Fresh tuber tissue (approx. 1 g) were homogenized in 5 cm^3^ of 80% methanol at 4 °C. Samples were centrifuged for 15 min (25 000 ×g, 4 °C). For total phenols, 0.1 cm^3^ of extract was mixed with 0.2 cm^3^ of Folin’s reagent (Sigma-Aldrich Chemie, GmBH, Steinheim, Germany) and 1.6 cm^3^ of 5% Na_2_CO_3_ and after 20 min incubation in 40 °C, the absorbance of samples was measured at 750 nm using Unico 2804 UV spectrophotometer. Using calibration curves made for gallic acid, the content of total phenols was calculated and expressed as gram per 1 kg of fresh weight tissue.

Radical scavenging activity was determined in tuber tissues (periderm, core and pith fragments). A stable free radical DPPH (2.2-diphenyl-1-picrylhydrazyl) was used to test the radical scavenging activity of extracts^26^. The changes in the absorbance of the DPPH solution, following the reduction of DPPH, were measured at 517 nm using Unico 2804 UV spectrophotometer. For the analysis, the same 80% methanol extracts were used for the phenolic compound analysis (see above). In the samples containing 2.9 cm^3^ 0.1 mM DPPH solution in 96% ethanol and 0.1 cm^3^ of extracts, an absorbance decrease of the DPPH solution was detected after 15 min. The control was prepared with 80% methanol instead of tuber part extract. The results were expressed as the percentage of DPPH neutralization using the fallowing equation:

$$\:\text{R}\text{a}\text{d}\text{i}\text{c}\text{a}\text{l}\:\text{s}\text{c}\text{a}\text{v}\text{e}\text{n}\text{g}\text{i}\text{n}\text{g}\:\text{a}\text{c}\text{t}\text{i}\text{v}\text{i}\text{t}\text{y}\:=\frac{\text{A}\text{b}\text{s}\text{o}\text{r}\text{b}\text{a}\text{n}\text{c}\text{e}\:\text{c}\text{o}\text{n}\text{t}\text{r}\text{o}\text{l}-\text{A}\text{b}\text{s}\text{o}\text{r}\text{b}\text{a}\text{n}\text{c}\text{e}\:\text{s}\text{a}\text{m}\text{p}\text{l}\text{e}}{\text{A}\text{b}\text{s}\text{o}\text{r}\text{b}\text{a}\text{n}\text{c}\text{e}\:\text{c}\text{o}\text{n}\text{t}\text{r}\text{o}\text{l}}\:x\:100\%$$

##### Estimation of lipid peroxidation level

Lipid peroxidation level was estimated using malondialdehyde (MDA) content method^27^. 1.5 g of fresh plant material was extracted with 6 cm^3^ of 0.1% trichloroacetic acid (TCA) at 4 °C and centrifuged for 15 min (25 155×g, 4 °C). Reaction mixtures (0.5 cm^3^ of extract and 0.5 cm^3^ of 0.5% thiobarbituric acid (TBA) in 20% TCA solution) were incubated for 30 min at 95 °C, cooled on ice and centrifuged for 10 min (25 155×g, 4 °C). The absorbance of the samples was measured at 532 nm and 600 nm using Unico 2804 UV spectrophotometer. MDA content was calculated according to Dhindsa et al.^27^ using MDA extinction coefficient (ε = 155 mM·cm^–1^). The value of absorbance for reaction mixture at 532 nm was reduced by correction value obtained at 600 nm. The results were expressed as mM of MDA per 1 kg of f.w.

### Statistical analysis

All analyses were made in three replications. The significant differences between arithmetic means were determined using Duncan test at *P* < 0.05. Statistica 12.0 software (StatSoft Inc. Tulsa, OK, USA) was used to conduct statistical analyses. The data were subjected to principal component analysis (PCA) performed for all analyzed traits in the pith, core and periderm in R v 4.4.1^28^ with the prcomp() function. The results were visualized for the first and second principal component with the fviz_pca_var() function from the factoextra v. 1.0.7.

## Results

### Size distribution analysis

SEM image analysis (see Supplementary material Fig. 1 for exemplary images) for the freshly prepared NPs samples indicates size distribution between 10 and 20 nm for synthesis with citrate and 15–25 nm with SDS. Samples, after 6 months of storage in a dark, air-conditioned environment (23 °C), exhibited only limited agglomeration. In the case of citrate, the size distribution shifted to 15–30 nm, and for SDS stabilizing agent to 25–60.

**Fig. 2:**
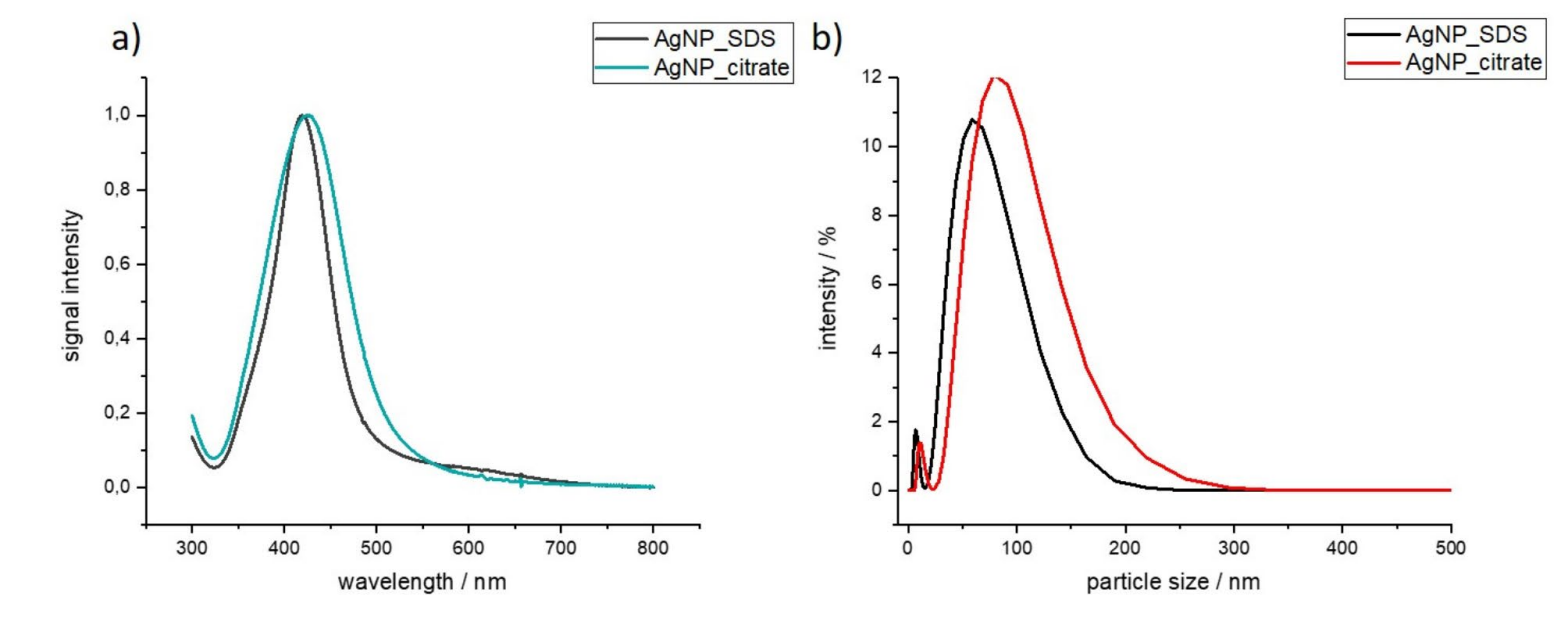
AgNPs size measurements: (**a**) UV-Vis absorption spectra of AgNPs prepared with SDS and citrate as capping agents; (**b**) DLS signal intensity and size distribution of AgNP_SDS and AgNP_citrate.

The UV-Vis spectra analysis (Fig. 2a) shows absorption peak at 420 and 435 nm for: SDS and citrate types of AgNPs, respectively. To measure nanoparticle sizes, the DLS methods, suitable for model spherical particle size distribution, was used. DLS measures hydrodynamic diameter, including both metal core and capping agent layer. Based on the DLS signal intensity the AgNP_citr samples consisted of both small individual nanoparticles (~ 15 nm) and larger conglomerates (~ 100 nm), the latter dominating signal intensity and increasing average size, yet not being the most numerous ones. Similar results were obtained for the AgNP_SDS sample series. Differences between each synthetic series are due to particle size polydispersion and existence in the samples of non-spherical particles/agglomerates which do not fit into model used for DLS analysis. Exemplary size distributions for one series of choice per each synthetic method are provided in Fig. 2b.

### Ag distribution in potato shoots and tubers

Analysis of silver ion concentrations in different parts of the potato shoots showed that spraying with the highest concentration of AgNPs_citr, led to the highest accumulation of silver in young leaves. At the same time silver accumulation in mature leaves and subsequent internodes was several dozen times lower (Fig. 1a-b). Spraying with the highest concentration of AgNPs_SDS, led to the highest silver accumulation in young leaves. However, in mature leaves, the seventh, fifth, third and first internode, silver ion accumulation was lower by approximately 2.5; 7; 5 and 8 times, respectively (Fig. 1a-b). Spraying with lower concentrations (1 and 0.1 mg·dm^−3^) of AgNPs_citr led to higher silver ion accumulation in leaves and younger internodes (0.20 to 0.36 mg·kg^−1^ d.w.) and much lower in older (lower ones) internodes (0.08 to 0.22 mg·kg^−1^ d.w.) (Fig. 1a-b). In contrast, spraying with lower concentrations of AgNPs_SDS resulted in the highest accumulation of silver ions in mature leaves and the lowest in young leaves (Fig. 1a-b).

In potato tubers, silver ion content ranging from 0.01 to 0.74 mg·kg^−1^ d.w. was recorded, depending on the AgNPs solution used and the part of the tuber (Fig. 1c). Spraying with AgNPs_citr_10 led to the accumulation of the highest Ag ion concentrations in the tuber pith and the lowest in the core (Fig. 1c). In contrast, spraying with AgNPs_SDS_10 resulted in the highest accumulation of silver in the tuber periderm (Fig. 1c). However, spraying with lower concentrations of AgNPs (0.1 and 1 mg·dm^−3^) led to accumulation of very low amounts of Ag (less than 0.1 mg·kg^−1^ d.w.) regardless of the part of the tuber, except in the tuber core of plants sprayed with AgNPs_citr_1 (Fig. 1c).

When comparing silver ion accumulation in individual organs depending on the applied spray, in young leaves, the most silver was recorded in plants sprayed with the highest concentrations of AgNPs_citr (Fig. 1a-b). In mature leaves, the most Ag ions were accumulated in plants sprayed with higher concentrations of AgNPs_SDS (Fig. 1a-b). Also at individual nodes, the most silver was accumulated after spraying with AgNPs_SDS_10 (Fig. 1a-b). In contrast, in individual parts of the potato tuber, the periderm, core and pith, silver was accumulated in significantly highest concentrations after spraying with citrate-synthesised nanoparticles at concentrations of 10 and 1 mg·dm^−3^ (Fig. 1a-b).

### Elements content in potato tubers

**Fig. 3:**
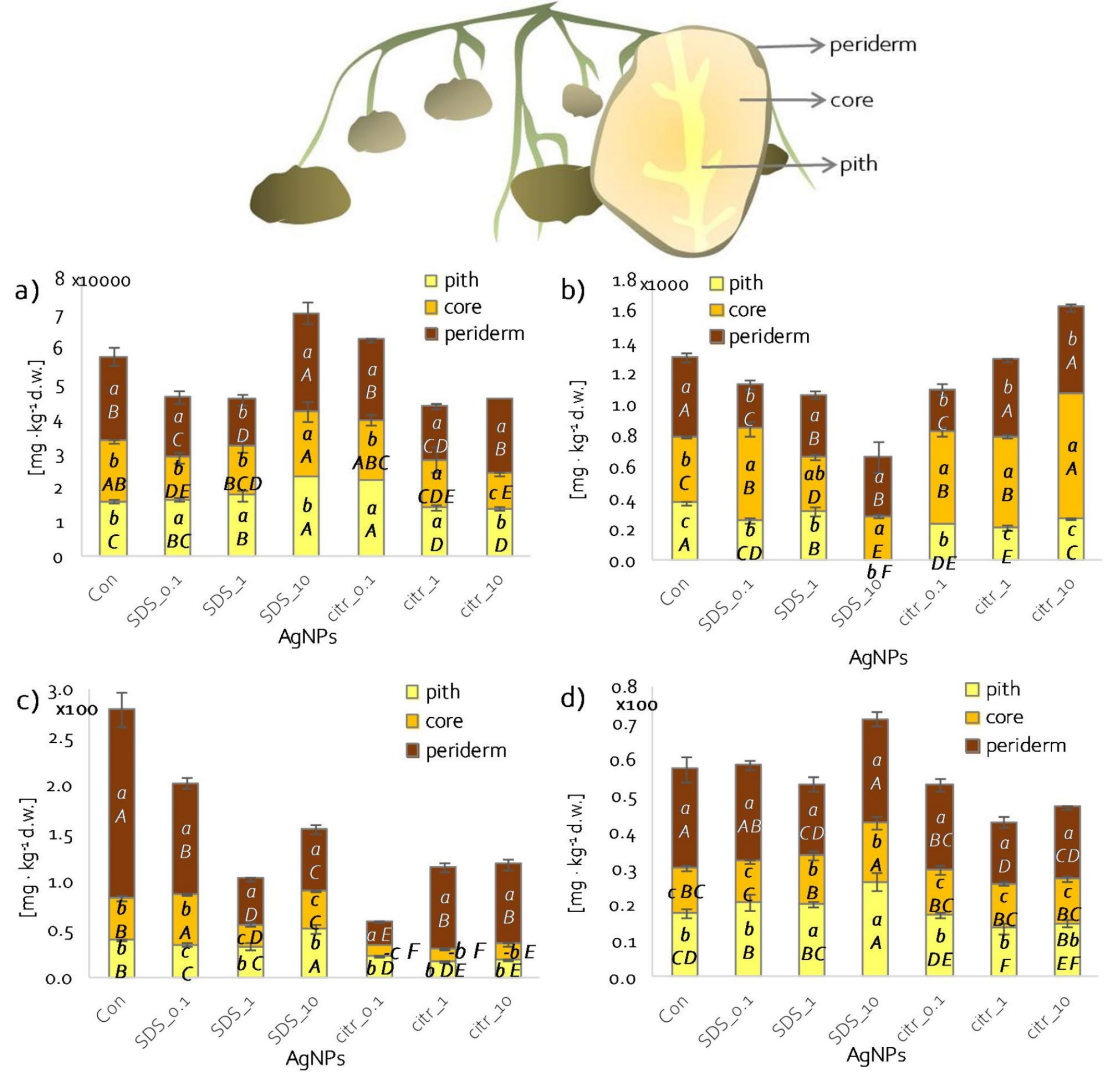
Content of: (**a**) K^+^; (**b**) Na^+^; (**c**) Fe^2+^ and (**d**) Zn^2+^ ions in periderm, core and pith of potato tubers depending on applied spray solution; different lowercase letters – statistically significant differences between tuber parts within single spraying solution at p ≤ 0.05; different uppercase letters - statistically significant differences between spraying solutions within single tuber part at p ≤ 0.05; n = 3.

The potassium content in tubers from unsprayed plants was highest in the periderm and at the same level in the core and pith (Fig. 3a). A similar distribution of potassium content was recorded in tubers from plants sprayed with the highest concentration of AgNPs synthesised with both SDS and citrate (Fig. 3a). In contrast, spraying with lower concentrations of nanoparticles synthesised with both SDS and citrate led to an increase in potassium content in the tuber pith relative to the core (Fig. 3a). The sodium content of unsprayed tubers was the highest in the periderm and the lowest in the pith (Fig. 3b). Spraying with citrate-synthesised nanoparticles (regardless of the concentration used) resulted in a significant increase in sodium accumulation in the tuber core relative to the periderm (Fig. 3b). A similar effect was observed in tubers of plants sprayed with the lowest concentration of nanoparticles synthesised with SDS (Fig. 3b). At higher concentrations of these nanoparticles, an increase in the sodium content of the tuber core to that of the periderm was observed (Fig. 3b).

The elemental content of the different parts of the tubers was also compared depending on the spray applied compared to the control (Fig. 3a-b *different uppercase letters*). In the case of potassium, the highest potassium content in the tuber periderm was recorded in plants sprayed with AgNPs_SDS_10, and significantly less, relative to the control, after spraying with lower concentrations of AgNPs_SDS and citr_1 (Fig. 3a). In contrast, in the tuber core and pith, spraying with AgNPs_citr_10 and _1 led to a decrease in potassium accumulation relative to the control. In the core, spraying AgNPs_SDS_10 and _1 led to a significant increase in K^+^ ion content relative to the control (Fig. 3a). As for sodium ions, both in the periderm, core and pith, their content decreased after spraying the plants with SDS-synthesized nanoparticles, especially at higher concentrations, relative to the control (Fig. 3b). Spraying AgNPs synthesized with citrate, increased sodium accumulation in the tuber core but decreased in the pith relative to the control (Fig. 3b).

Spraying with nanoparticles generally caused a decrease in iron ion content in all parts of the tubers compared to control plant tubers (Fig. 3c). However, this decrease was greater in tubers (especially core and pith) of plants sprayed with nanoparticles synthesized with citrate than with SDS (Fig. 3c). The iron ion content in tubers of control plants was highest in the periderm and at the same level in the core and pith (Fig. 3c). Spraying with SDS-synthesised silver nanoparticles changed the level of Fe^2+^ accumulation in the core and pith of tubers depending on the used AgNPs concentration (Fig. 3c). In the pith, Fe^2+^ content decreased when plants were sprayed with AgNPs_SDS_0.1, and in the core when plants were sprayed with higher concentrations (1 and 10 mg·dm^−3^) (Fig. 3c). In the case of citrate-synthesised nanoparticles, only a concentration of 0.1 mg·dm^−3^ resulted in a decrease in iron content in the tuber core (Fig. 3c). However, spraying with SDS-synthesized silver nanoparticles increased the total Fe content in the pulp (core plus pith) relative to the periderm and in some cases even exceeding it (Fig. 3c).

In the described experiments, in the tubers of control plants, the highest zinc content was recorded in the periderm and the lowest in the core (Fig. 3d). Spraying the plants with citrate-synthesised silver nanoparticles did not change the distribution of zinc accumulation in the different parts of the tuber (Fig. 3d). In contrast, spraying with medium and highest concentrations (1 and 10 mg·dm^−3^) of SDS-synthesised nanoparticles increased zinc accumulation in the tuber pith to the level as in the periderm (Fig. 3d). However, the total zinc content of the tuber core and pith exceeded that of the periderm. Nevertheless, the zinc content of the periderm constituted on average approximately 40% of the zinc content in the whole tuber. However, spraying with Ag nanoparticles modified the Zn^2+^ content of different parts of the tuber compared to the control. The zinc content in the periderm and pith of tubers decreased significantly under the spraying of citrate-synthesized nanoparticles compared to the control (Fig. 3d). In contrast, spraying with the highest concentration of AgNPs_SDS resulted in an increase in zinc content in the core and pith of tubers relative to the control (Fig. 3d).

### LAA and sugar content of potato tubers

**Fig. 4:**
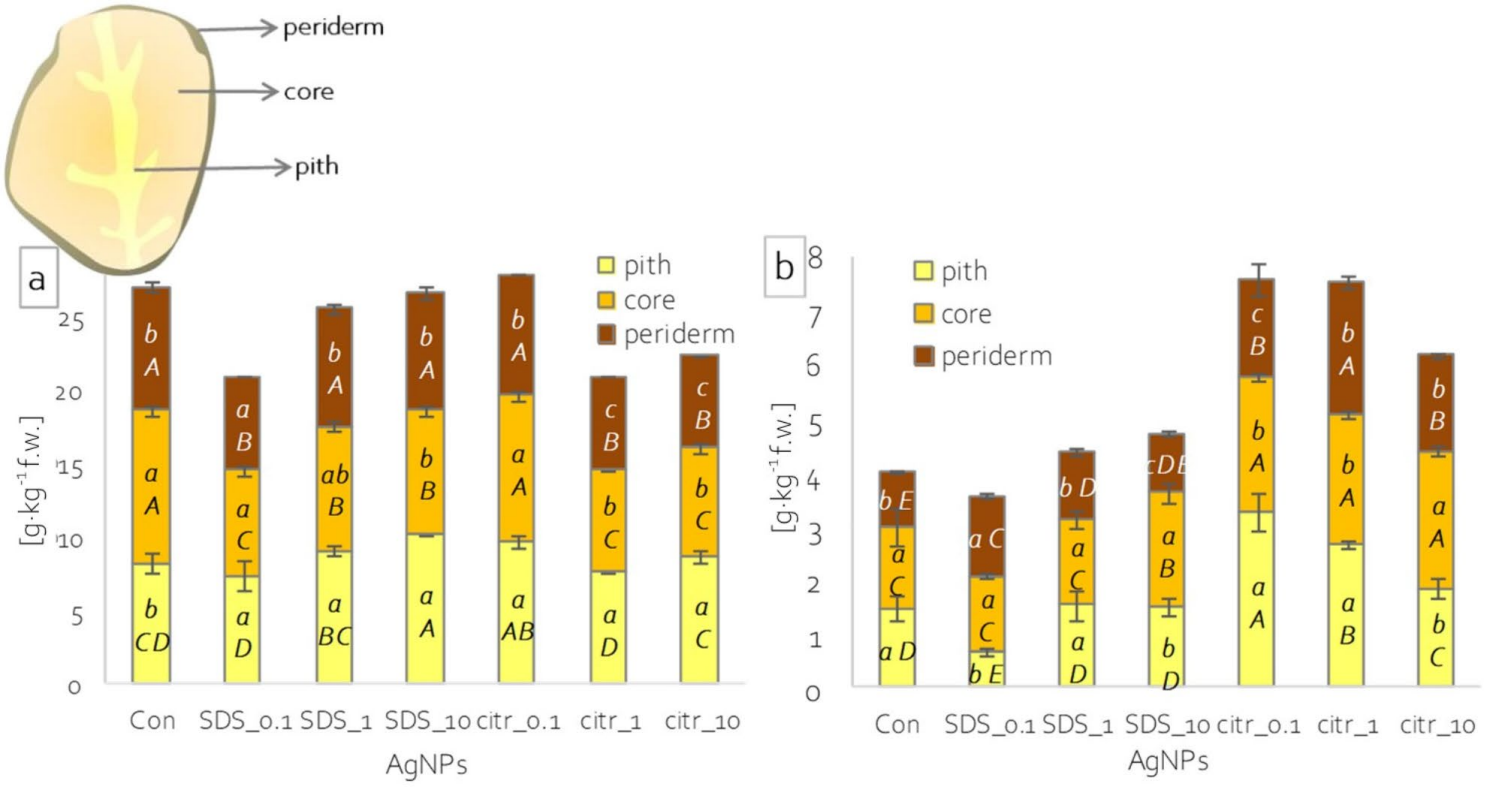
Composition of potato tubers: (**a**) L-ascorbic acid content; (**b**) soluble sugar content in periderm, core and pith of potato tubers depending on applied spray solution; different lowercase letters – statistically significant differences between tuber parts within single spraying solution at p ≤ 0.05; different uppercase letters - statistically significant differences between spraying solutions within single tuber part at p ≤ 0.05; n = 3.

L-ascorbic acid content in tubers of control plants was highest in the core. Meanwhile, spraying the plants with silver nanoparticles increased the LAA content in the tuber pith relative to the periderm, while spraying AgNPs_citr_1 and _10 resulted in a decrease in the core relative to the pith (Fig. 4a). On the other hand, comparing the LAA content in different parts of the tubers depending on the spray applied, the application of higher concentrations of AgNPs_citr led to a significant decrease in LAA content in the tuber periderm and core compared to unsprayed plant tubers (Fig. 4a). In contrast, spraying with SDS-synthesized Ag nanoparticles led to a decrease in LAA in the core, while the highest concentration of Ag nanoparticles led to an increase in LAA content in the pith (Fig. 4a).

The content of soluble sugars in the tubers of unsprayed (control) plants was lower in the periderm, and higher and at the same level in the core and pith (Fig. 4b). Spraying with silver nanoparticles caused changes in such content distribution. The highest concentrations of AgNPs, both synthesized with SDS and citrate, led to a decrease in sugar content in the pith relative to the core (Fig. 4b). Spraying with the other AgNPs_citr concentrations (1 and 0.1 mg·dm^−3^), on the other hand, led to a decrease in sugars in the core compared to the pith of the tubers (Fig. 4b). In general, spraying plants with Ag nanoparticles synthesized with citrate resulted in an increase in sugar content in all parts of the tubers relative to the control (Fig. 4b). In contrast, spraying the plants with AgNPs_SDS, at most of the concentrations used, did not change the sugar content of the various tuber parts, with a few exceptions (Fig. 4b).

### Antioxidant properties of potato tubers

**Fig. 5:**
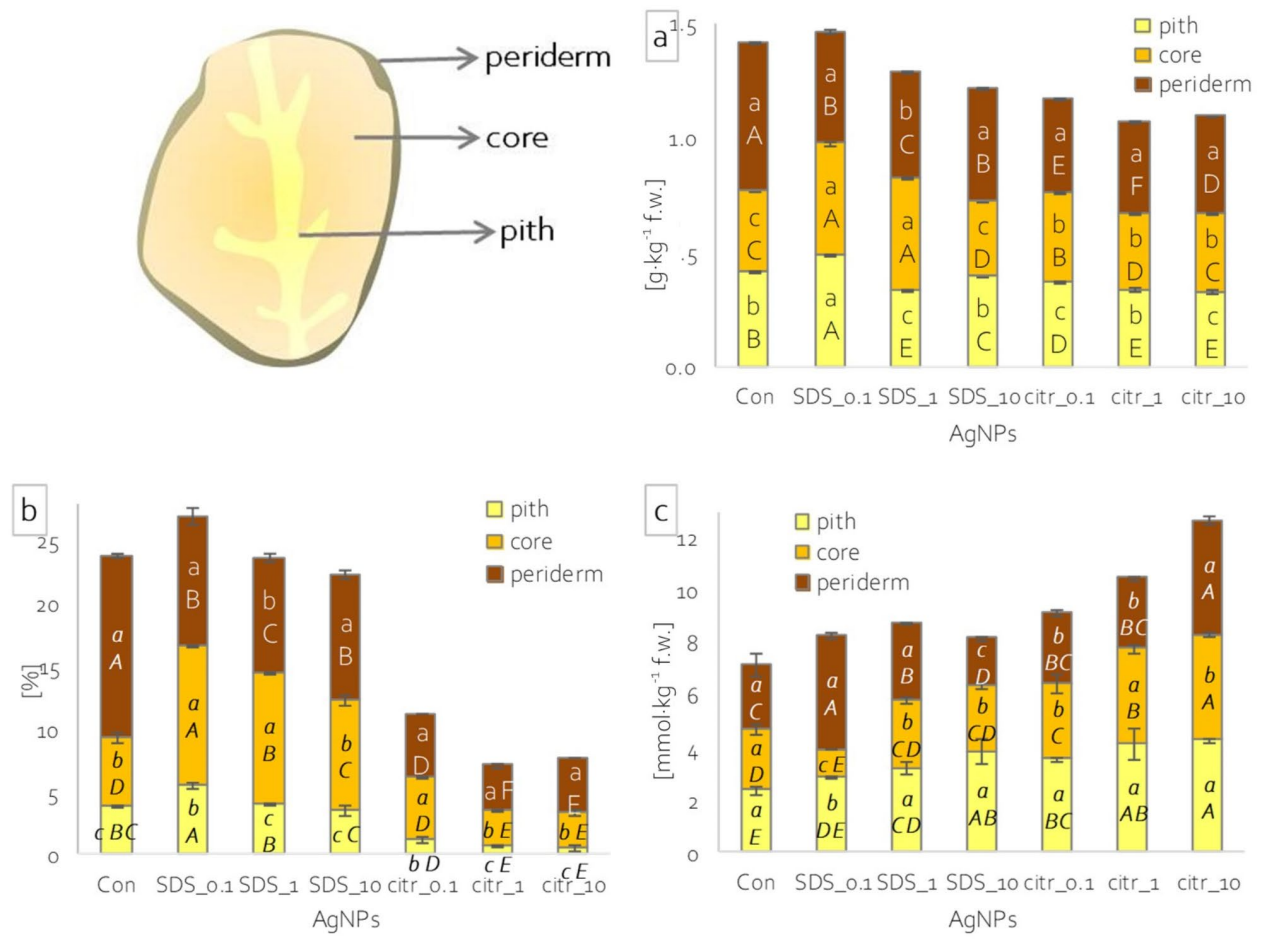
Antioxidant properties of potato tuber: (**a**) phenols content, (**b**) radical scavenging activity and (**c**) lipid peroxidation level in periderm, core and pith of potato tubers depending on applied spraying solution; different lowercase letters – statistically significant differences between tuber parts within single spraying solution at p ≤ 0.05; different uppercase letters - statistically significant differences between spraying solutions within single tuber part at p ≤ 0.05; n = 3.

Examination of lipid peroxidation levels showed no differences between different parts of tubers in control plants (Fig. 5c). In contrast, spraying with silver nanoparticles led to significant changes in lipid peroxidation levels, both between different parts of the tubers and between solutions relative to the control. Spraying with all concentrations of AgNPs_citr increased lipid peroxidation in the tuber pith relative to the other parts, while spraying with higher concentrations also increased lipid peroxidation in the core or periderm, depending on the concentration (Fig. 5c). It also caused an increase in lipid peroxidation relative to unsprayed plants, especially in the core and pith (Fig. 5c). Whereas spraying with AgNPs_SDS reduced lipid peroxidation in the core relative to the pith, and lower concentrations also reduced lipid peroxidation relative to the periderm. The highest concentration of AgNPs_SDS also reduced lipid peroxidation in the periderm relative to the core and pith (Fig. 5c). In contrast, spraying with these nanoparticles did not change the level of lipid peroxidation relative to the control in the core, but increased it in the pith and periderm, with a few exceptions (Fig. 5c).

The content of phenolic compounds in the control plant tubers was the highest in the periderm and the lowest in the core. Spraying plants with citrate-synthesized silver nanoparticles increased phenolic content in the core relative to the tuber pith (Fig. 5a). In contrast, when plants were sprayed with AgNPs_SDS, only the lower concentrations caused a change in phenolic accumulation in tubers. The lowest concentration applied (AgNPs_SDS_0.1) increased phenolic content in both the core and the pith to a level as in the periderm. Meanwhile, the intermediate concentration (AgNPs_SDS_1) increased phenolic content in the core relative to the pith and periderm, and decreased in the pith relative to the core (Fig. 5a). On the other hand, comparing the effect of the solutions in the different parts of the tubers against the control, a decrease in the phenolic content in the periderm was noted after the spraying of nanoparticles regardless of the synthesis method. However, the decrease was greater after spraying AgNPs synthesized with citrate than with SDS (Fig. 5a). A decrease in phenolic content was also observed in the core and pith of plant tubers sprayed with nanoparticles (Fig. 5a). The exception was a core and pith of plant tubers sprayed with lower concentrations of AgNPs_SDS, where the content of phenolic compounds increased compared to the control (Fig. 5a).

In turn, the ability (activity) to scavenge free radicals, determined by the method using DPPH, in control tubers was highest in the periderm, lower in the core and the lowest in the pith (Fig. 5b). The application of citrate-synthesized silver nanoparticle spraying did not change the distribution of free radical scavenging capacity among the different parts of the tubers, but led to its decrease relative to the control in all parts of the tuber (Fig. 5b). For spraying with AgNPs_SDS it was recorded similar changes as for phenolic content (Fig. 5b).

### Principal component biplot analysis

**Fig. 6:**
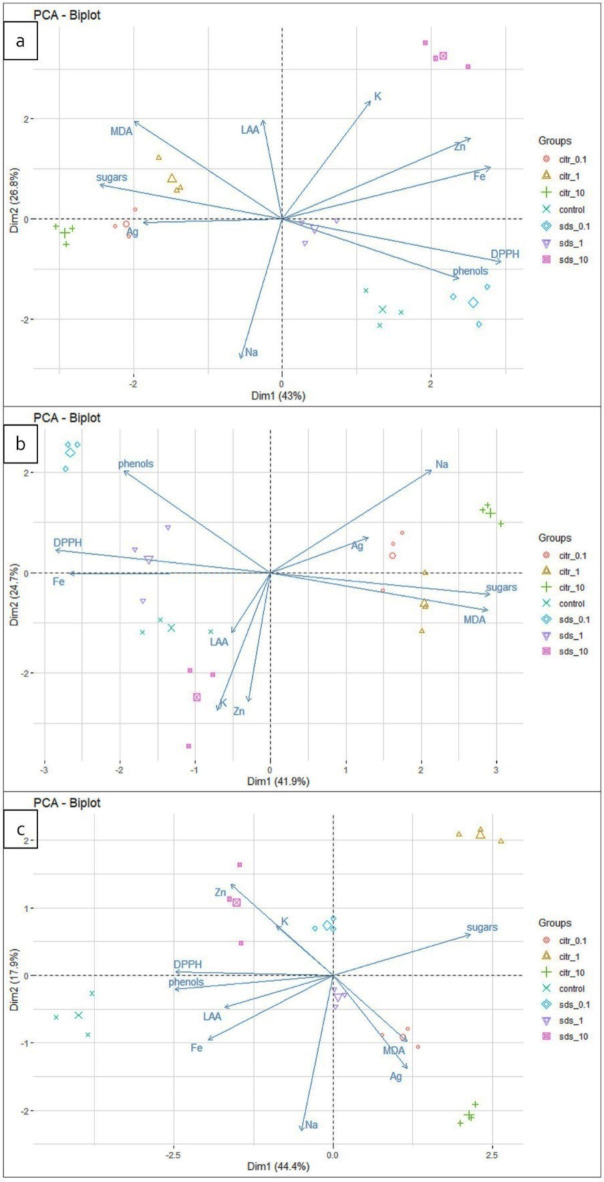
Principal component analysis biplots showing relationships between the AgNPs spraying and nutritional parameters of the pith (**a**), core (**b**) and periderm (**c**) of the potato tuber.

PCA biplots were generated for tuber pith, core and periderm separately (Fig. 6). Biplots indicated that in tuber pith both principal components explained almost 70% of the total variance, with the first principal component (Dim1) explaining 43%, and the second (Dim2) 26.8% (Fig. 6a). The analysis revealed clear differences in tuber pith responses depending on AgNPs type used, as distinguished by Dim1. Spraying with lower concentration of AgNPs_SDS resulted in similar pith reaction as control, while the highest concentration triggered a different response as distinguished by Dim2. While, after spraying with AgNPs_citr reaction of tuber pith was rather similar, regardless of the concentration. The PCA biplots explained also almost 67% (Dim1: 41.9%, Dim2: 24.7%) of the total variance in the tuber core (Fig. 6b). As with the tuber pith, the tuber core exhibited differential reactivity to silver nanoparticles synthesized by various methods, as discriminated by Dim1 and to the lowest and the highest AgNPs_SDS concentration, as distinguished by Dim2. Regarding the periderm, PCA biplots explained 62.3% (Dim1: 44.4%, Dim2: 17.9) of the total variance (Fig. 6c). Clear differences was observed in response of periderm between control and nanoparticle treatments (discerned by Dim1 and Dim2). Biplots indicated also differences in the response of the periderm to the nanoparticle concentrations used, as explained by the second principal component (Dim2).

Furthermore, the analysis revealed that potato pith reacted to AgNPs_SDS spraying with increase in the content of K^+^, Zn^2+^, Fe^2+^ ions and phenols as well as antiradical activity. While spraying with AgNPs_citr was associated with an increase in sugars and Ag^+^ ion content content as well as lipid peroxidation levels (Fig. 6a).

In tuber core, after spraying with the highest AgNPs_SDS concentration, increased considerably content of potassium and zinc and slightly content of LAA. While, reaction of core to lower AgNPs_SDS concentration was accompanied by enhance phenols content and antioxidant capacity. In turn, in tuber core, after application of AgNPs_citr, increase of sugar, MDA and Ag^+^ ion content was evidenced (Fig. 6b). By comparison, the periderm displayed an increase in K^+^ and Zn^2+^ content, along with a slight enhancement in phenol and antiradical activity following the application of AgNPs_SDS_10. In contrast, after spraying with AgNPs_citr, the periderm exhibited a notable increase in silver content and lipid peroxidation levels (Fig. 6c).

For all parts of tubers, PCA biplots revealed positive correlation between potassium and zinc content, between phenols and antioxidant capacity, between MDA, sugar and silver content and negative correlation between silver and potassium content, between silver and zinc content, between antioxidant capacity, phenols content and sugar, lipid peroxidation level (Fig. 6).

## Discussion

AgNPs trigger a range of anatomical-morphological and biochemical-physiological reactions in plants, generating positive or negative effect. These effects depend on the size, concentration, duration of action, and surface coating type of nanoparticles, application site and method as well as species and plant vegetative phase^29^. Most often, NPs are applied in water/medium and applied to the substrate leading to the highest accumulation of nanoparticles in plant root and negatively affecting the rhizosphere microorganisms^30^. Meanwhile, nanoparticle application by spraying produces monodisperse particles and prevents their agglomeration^31^. In this form, NPs retain their dimensions, which determines their effective penetration through stomata^32,33^.

The physical and chemical properties of nanomaterials depend on particle size^34^. The size and shape of metal nanoparticles can be controlled by the choice of stabilizer^34^, in the presented study using SDS or citrate. The UV-Vis absorbance maxima obtained for AgNP_citr and AgNP_SDS corresponded to diameters of nanocrystalline silver core ranging between 10 and 50 nm^11^, respectively. Differences in the size of nanoparticles obtained by the two methods (with citrate and SDS) were also demonstrated by DLS and SEM image analysis. In addition, SEM analysis showed that the nanoparticles stored for 6 months agglomerated to a small extent, aligning well with known nanoparticle stabilization strategies^35^. Absorbed nanoparticles are basipetal transported, depending on their size, by apoplastic pathway, when the NPs size is about 200 nm, or by symplastic pathway, when NPs size is equal or smaller than 50 nm^36^. In the case of potato, nanoparticle size ranged from 15 to 126 nm depending on the synthesis method so they could be transported both apoplastic and symplastic ways.

Since silver ions in excess can pose a potential risk to humans, it is important to examine the distribution of Ag ions in the sprayed plants, especially in the edible parts, and thus assess the nutritional risk^37^. Most silver ions were accumulated in the potato shoots, especially in leaves and younger internodes, reflecting the application method. However, it was observed that SDS as a nanoparticle stabilizer reduced the mobility of silver in the plant, which was associated with the larger size of these nanoparticles. The study also revealed that despite the potential for apoplastic and symplastic transport, the concentration of Ag ions in potato tubers was low, especially in the case of spraying with AgNPs synthesized with SDS (Fig. 1c). Significantly, in the tubers, most silver ions were accumulated in the periderm, that is usually discarded before consumption^38^. Nevertheless, considering that the silver ingestion limit is 5 µg·kg^−1^·day^−1^ (about 350 µg·day^−1^ for an average adult weighing about 70 kg)^37^, the recorded Ag content in potato tubers does not pose a nutritional risk.

Potato tubers are an excellent source of minerals which are essential for the proper functioning of the human body^38,39^. Furthermore, they demonstrate high bioavailability of minerals due to a high content of substances that enhance the absorption of essential microelements^39^ and a low content of anti-nutritional factors^40^. Therefore, assessing the mineral content of tubers is an important part of determining their consumption values. In the collected potato tubers, the content of chosen macro- and microelements was examined in different parts depending on the spray applied. Among the macroelements present in potatoes, potassium (K^+^) content was examined as the element most abundant in the tubers and crucial for potato’s health-promoting properties, such as regulation of heart function and lowering blood pressure^41^. Whereas, sodium (Na^+^) content, although occurring at relatively low levels in potato tubers, was analysed because a high-sodium diet causes increased blood pressure and hypertension^41^. Moreover, in humans, sodium and potassium regulate and control total electrolyte management, participate in the body’s acid-alkaline balance, and serve a major role in the stimulus conduction in all nerve cells^42^. Recommended Dietary Allowance (RDA (US)) of potassium and sodium is 4700 mg and 1500 mg, respectively (for adults age 31 to 50)^43^, and 100 g of boiled potatoes can provide up to 16% of the Adequate Intake (AI) of potassium^41^. In the present study, potassium content was higher in pulp (total content in core and pith) than in peel (periderm) of potato tubers. In contrast, other studies observed about two times higher K^+^ content in peel than in pulp^38^. The sodium content of potato tubers in the studies described was very high, ranging from about 200 to 800 mg·kg^−1^, depending on the part of the tuber and the spray applied. In comparison in the studies of other authors, the values for Na^+^ content for ‘Tajfun’ variety varied between about 220 and 360 mg·kg^−1^ d.w., depending on the year and cultivation method^44^. Interestingly, the content of Na^+^ ions in the pulp (core and pith) of control tubers was almost 800 mg·kg^−1^ but in the pulp of AgNPs_SDS_1 and 10 sprayed tubers was lower, approximately 660 and 280 mg·kg^−1^, respectively.

Equally important as macroelements, in the human diet, are microelements, that, performing structural and functional roles, serving as an integral part of many enzymes and regulating metabolism, ensure the proper functioning of the body^38^. In the present study, zinc and iron were determined because their content in potato tubers is the highest among micronutrients. The contents of iron and zinc in raw potatoes range from 2.5 to 8.3 mg·kg^−1^ f.w. and 2.3 to 3.9 mg·kg^−1^ f.w., respectively^41^. Moreover, microelements deficiencies are common in both developing and developed countries. To give an example, it is estimated that 60% of the current world population suffers from iron deficiencies and 30% from zinc deficiencies^38,39^. RDA (US) for iron and zinc, amounts 18 and 11 mg, respectively^43^. According to Burgos et al.^41^, content of iron and zinc in 100 g of cooked potatoes ranges between 0.29 and 0.69 and 0.29–0.48 mg, respectively, covering 1.6–3.8 and 2.6–4.3% recommended dietary allowance, respectively. In presented study, content of Fe^2+^ and Zn^2+^ was the highest in periderm. Several times higher content of these elements in peels (periderm) compared to pulp was also observed by other authors^38,40^. However, the pulp (core and pith) from tubers of plants sprayed with AgNPs_SDS_10 contained more of these elements than peels.

Mineral nutrients are taken up by plants mainly from the soil solution through the roots. Their redistribution into the tuber, happens through the phloem from the aboveground parts of the plant^40^. The translocation of mineral nutrients and their accumulation in the underground organs of the plant are influenced by both environmental and genetic factors, including the availability of nutrients in the soil, the anatomy of the tuber, the mechanisms responsible for: transport and sequestration within the organ, loading and unloading of phloem and xylem, or transfer through the periderm^39,40^. Our research indicates that spraying with silver nanoparticles modifies the distribution and storage patterns of some macro- and microelements in the potato tuber.

L-ascorbic acid (LAA, vitamin C) plays a pivotal role in human nutrition and health, including prevention of scurvy. For humans, who are unable to synthesize vitamin C, the principal sources of this vitamin are vegetables and fruits^45^. The potato is one of the most widely consumed vegetables in the world today. Vitamin C represents the most abundant vitamin in potatoes, with an average content range of 0.8–3 g·kg^−1^ fresh weight^45,46^. Estimated Average Requirement of vitamin C for adults ranges between 60 and 75 mg per day^47^. Assuming a loss of approximately 50% of vitamin C during the preparation of potatoes^41^, a portion of 100 g of cooked potatoes, containing on average 30 mg of vitamin C, can provide 20–25% of the Estimated Average Requirement (EAR). As potato consumption tends to be much higher than that of other vegetables, it can make a significant contribution to total dietary intake of vitamin C^41^. Furthermore, L-ascorbic acid is of significant importance as it enhances the bioavailability of iron, due to its properties that mitigate the chelating effect of phytic acid^46^. Prior research indicates that the vitamin C content of potatoes is subjected to influence from both genotype and growing conditions, including soil type and climate^45,46^. The vitamin C content in the tubers of the tested variety ranged from 210 to 271 mg/kg, about 70% of which was in the pulp (core plus pith). In general, the application of silver nanoparticles at higher concentrations resulted in either no alteration in the vitamin C content (AgNPs_SDS) or a reduction in it (AgNPs_citr) when compared with the control. Nevertheless, a modification in the accumulation pattern was identified in specific parts of the tubers. The concentration of vitamin C increased in the pith and decreased in the core. Such alterations may be associated with the plant’s reaction to the stres factor - silver ions. However, due to the minimal rate of nanoparticle penetration into the tubers (i.e., modified underground shoots), LAA accumulates around the vascular bundles, as the rapid translocation to other plant parts is unnecessary^48,49^.

The concentration of sugars in potato tubers is a significant determinant of the quality of potatoes. High content of soluble sugars is not desirable in potato tubers. It is due to their properties, which lead to non-enzymatic browning, resulting in a reduction of tuber quality^50^. The level of sugars in potato tubers is affected by a number of factors, including genotype, environmental conditions and cultivation methods during growth, as well as post-harvest handling and storage^51^. It was recorded between approximately 100 and 150 g·kg^−1^ f.w. of soluble sugars in the tubers of the tested potato variety in control conditions. While, Pszczółkowski et al.^52^, for the same cultivar, recorded a sugar content between 40 and 83 g·kg^−1^ f.w. The elevated sugar content observed in the control tubers may be attributed to the processing methodology employed, which involved fragmentation of the tubers. Kumar et al.^51^ observed that mechanical treatment of tubers, particularly the removal of the periderm, resulted in an increase in sugar content. However, spraying with silver nanoparticles, especially those synthesised with citrate, altered the sugar content of the tubers, increasing their levels and altering their accumulation pattern in different parts of the tuber. Sugars are involved in plant growth and development as: structural components, energy sources for various metabolic activities, reserves and as signalling molecules in signal transduction pathways^53^. Given their diverse roles, an increase in the content and translocation of sugars within tubers may be linked to growth processes and also contribute to the immune response^54^ to an emerging stress factor such as Ag ions.

A comparative analysis of the antioxidant activity of various vegetables demonstrated that the potato exhibits relatively low antioxidant activity^55^. It should be noted, however, that potatoes have a relatively high consumption rate when compared with other vegetables. Consequently, a minor increase of the antioxidant activity and phenolic compounds content of potato tubers results in a proportional increase in the intake of bioactive compounds in the diet^55^. Phenolic content and antioxidant activity are influenced primarily by genotype^55^, but also by environment and growing conditions^56,57^. Furthermore, studies conducted on in vitro potato explants treated with silver nanoparticles demonstrated an increase in the level of lipid peroxidation, indicating an increase of oxidative stress^58^. Increased oxidative stress will lead to the activation of antioxidant mechanisms in the plant and increase, among others, the content of phenolic compounds. It is noteworthy that elevated antioxidant activity, in addition to strengthening the health-promoting properties of potato tubers, can enhance protection against pathogenic microorganisms during cultivation and tuber storage^59^. Consequently, it was reasonable to test whether spraying with silver nanoparticles would affect the degree of lipid peroxidation, content of phenolic compounds and antioxidant activity in the tubers of tested potato cultivar.

The process of lipid peroxidation, which results in the formation of malondialdehyde, is induced by the activity of reactive oxygen species (ROS) generated by stress factors. The transport of AgNPs resulted in the release of silver ions, which are known to disrupt cellular functions and induce phytotoxicity by binding to cellular components and modulating their activity, and thus generating ROS^10^. During lipid peroxidation, components of cellular membranes and consequently the overall structure of the membrane are damaged. This results in increased membrane permeability, which subsequently hinders the proper functioning of cells^60^. If this process occurs in the edible parts of plants, it would result in a reduction in their nutritional value^61^. An increase in lipid peroxidation results in a reduction in the consumption values of potato tubers, due to a more pronounced browning of these tubers^62^. The presence or absence of lipid peroxidation, and related changes in MDA content, may serve as an indirect indicator of the overall functionality of the antioxidant system. The results of the present study showed that the degree of lipid peroxidation varied according to the type of nanoparticles used, their concentration and the part of the tuber. The greatest increase in malondialdehyde (MDA) content, and thus the highest level of oxidative stress, was observed following the application of AgNPs_citr. The distribution of MDA content in the different tuber sections indicated that oxidative stress was highest in the pith, suggesting that this part of the tuber was the most susceptible to the effects of Ag ions. This was also indirectly reflected in the increase in soluble sugar content.

Polyphenols represent a significant group of antioxidants and are regarded as the most prevalent antioxidants in human diet^57^. A review of literature revealed no information concerning the impact of silver nanoparticles on the phenolic compounds content and antioxidant activity of potato tubers. In contrast, in vitro studies demonstrated that the addition of silver nanoparticles to the culture medium altered the phenolic compounds content in 4-week-old potato plantlets^58^. Nevertheless, the content of phenolic compounds and antioxidant activity of potato tubers was the subject of analysis in field experiments conducted under different cultivation conditions^56,57^. The average phenolic content in potato tubers of different cultivars varied between 62.6–1157. mg∙kg^−1^ f. w, while an average of 155.0 mg∙kg^−1^ f. w^57^. , 169.1 mg∙kg^−1^ f. w^56^. and 712.7 mg∙kg^−1^ f. w^63^. was recorded in tubers of the cultivar ‘Tajfun’ in earlier studies. The present study revealed that the phenolic content of the tubers exhibited considerable variation, ranging from 330 to 650 mg∙kg^−1^ f.w. This variability was observed to be dependent on both the specific part of the tuber and the spray applied. As observed by Kim et al.^64^, the highest phenolic contents were found in the periderm of the tubers (400–650 mg∙kg^−1^ f.w.). Overall, the application of silver nanoparticles, with the exception of AgNPs_SDS_0.1, resulted in a reduction in the phenolic compound content across all tested tuber parts. This suggests the existence of a disparate mechanism of response to the stress factor.

Reactive oxygen species, generated as a by-product of metabolic reactions, are tightly linked to processes such as growth, environmental stress regulation, development and defense mechanisms^64^. Given the toxicity of these reactive molecules, research into the mitigating effects of radical scavengers has become a focus for the prevention of disease. One of the most frequently employed radicals for the assessment of antioxidant activity is the DPPH radical^64^, which was utilised in this study to quantify the radical scavenging activity of extracts derived from various parts of the tuber. Kim et al.^64^ demonstrated that the antioxidant capacity of potato tuber periderm and pulp extracts, prepared in 80% methanol, exhibited an average antioxidant activity of approximately 40% in both cases. In contrast, Im and Suh^65^ reported antioxidant activity of different potato cultivars at 6.9–14.6% in the tuber pulp and 9.2–18.3% in the periderm. The presented studies revealed that the antiradical activity exhibited the lowest levels in the pith (0.5–5.6%) and the highest levels in the periderm (3.6–14.6%) of the tested tubers. The application of Ag nanoparticles, similar to phenolic compounds, with the exception of AgNPs_SDS_0.1, resulted in a reduction of antiradical activity.

The results presented in current study indicate that foliar spraying of silver nanoparticles affected the nutritional properties of potato tubers. The synthetic method for nanoparticles determined the final distribution and accumulation of silver ions in the plant. Lower amounts of silver ions were transported to the underground parts of the potato (tubers) when synthesized with incorporation with SDS as capping agent, rather than with citrate. This method of synthesis was also more favourable in terms of nutritional properties of potato tubers. Spraying with the highest tested concentration of AgNPs_SDS had a favourable effect on the nutritional parameters of potato tubers including a variety of macro- and micronutrients, ascorbic acid and soluble sugars. On the other hand, lower concentrations of AgNPs_SDS improved the antioxidant properties of tubers, increasing the content of phenolic compounds and free radical scavenging efficiency. Based on these results further research is needed to verify if and how spraying with silver nanoparticles will affect the resistance of potato plants to pathogens and pests during cultivation, as well as affect tubers upon prolonged storage conditions.

## Electronic supplementary material

Below is the link to the electronic supplementary material.

Supplementary Material 1

## Data Availability

The data analysed during the current study available from the corresponding author on reasonable request.
