# Supplementary material for: Effect of silver nanoparticles foliar application on the nutritional properties of potato tubers
Source: Sci Rep. 2024 Sep 18;14:21753. doi: 10.1038/s41598-024-73096-2 (PMC11411065; doi:10.1038/s41598-024-73096-2)
Supplement: Supplementary file 1 — Supplementary Material 1 [file 41598_2024_73096_MOESM1_ESM.pdf]

**Supplementary Information**

# Effect of silver nanoparticles foliar application on the nutritional properties of potato tubers

Krzysztof M. TOKARZ[a*1.], Tomasz MAZUR[b1.], Monika HANULA[c], Wojciech MAKOWSKI[a], Piotr ZAWAL[b], Roman J. JĘDRZEJCZYK[d], Konrad SZACILOWSKI[b], Stanisław MAZUR[a], Wojciech WESOŁOWSKI[e], Barbara TOKARZ[a]*

Alongside DLS size measurements, additional SEM imaging took place, showing that upon time of several months nanoparticles from each synthesis have agglomerated. Each image represents nanoparticles drop casted from solution on Si wafer surface.

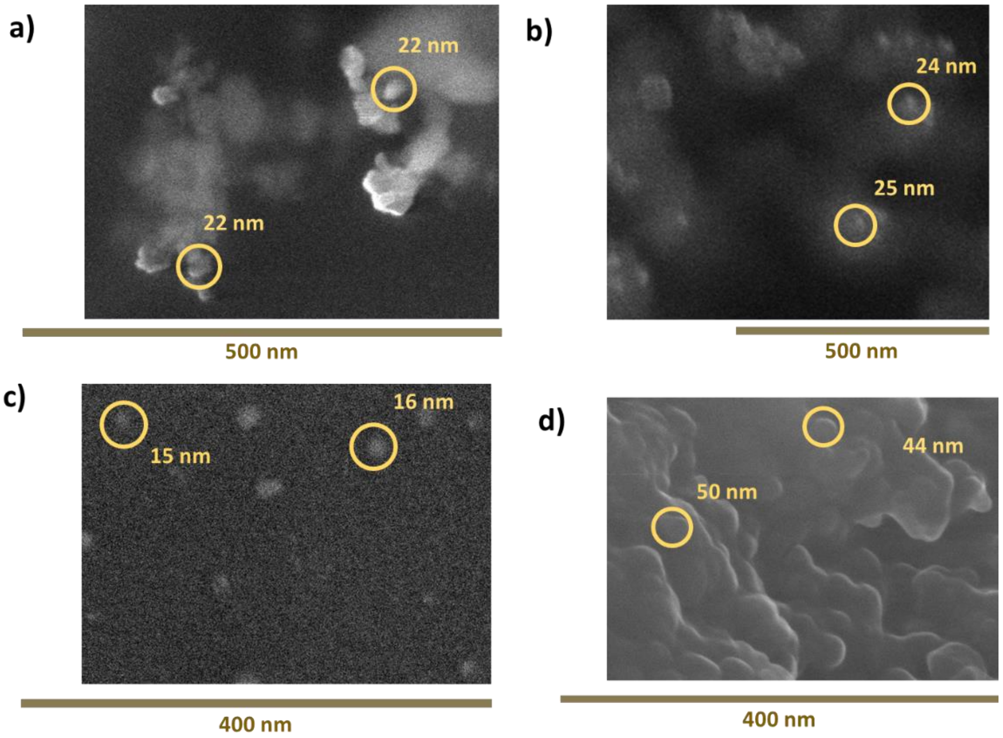


Figure 1. SEM images of Ag nanopartciles: a-b) AgNP_citrate directly after synthesis (a) and 6 months after synthesis (b); c-d) AgNP_SDS directly after synthesis (c) and 6 months after synthesis (d).
